# Constraints to maternal healthcare access among pastoral communities in the Darussalam area of Mudug region, Somalia “a qualitative study”

**DOI:** 10.3389/fpubh.2023.1210401

**Published:** 2023-09-14

**Authors:** Hodan A. Duale, Abdiqani Farah, Abdi Salad, Sumaya Gele, Abdi Gele

**Affiliations:** ^1^Department of Maternal and Reproductive Health, Somali Institute for Health Research, Hargeisa, Somalia; ^2^Faculty of Medicine, Al-Hayat Medical University, Mogadishu, Somalia; ^3^Faculty of Medicine, Somali National University, Mogadishu, Somalia; ^4^Faculty of Medicine, Wroclaw Medical University, Wroclaw, Poland; ^5^Department of Health Service Research, Norwegian Institute of Public Health, Oslo, Norway

**Keywords:** pastoralist, mother and child health, transhumant, maternal death, newborn, health care services

## Abstract

**Background:**

While countries embrace efforts to achieve Sustainable Development Goals (SDG) goal 3.1 (to reduce the global maternal mortality ratio to less than 70 per 100,000 live births by 2030 and end preventable deaths of new-borns and children), an estimated 2.5 million pastoralists in Somalia are struggling to access maternal and child healthcare services. Institutional delivery and access to antenatal care remained to be a challenge in Somalia, where pastoralism is a common means of livelihood. The aim of this study is to explore the maternal health services available for settled pastoralists (transhumant) and their families who still practice nomadic pastoralism in the Mudug region of Somalia.

**Methods:**

A qualitative study, including 14 interviews and one FGD, was conducted in Darussalam village (a transhumant village along the border between Somalia and Ethiopia), Puntland State, from December 2022 to January 2023. The study participants were community members who support the maternal and child health clinic (MCH), village administration, and health providers.

**Results:**

We found that the efficiency of the health facilities that serve for pastoralist women and children are hampered by staff-related, supply-related, patients-related and referral-related constraints. This study highlights that the absence of essential supplies, the unmet need for training among the staff as well as the absence of important facilities in the MCH such as ambulance and blood bags.

**Conclusion:**

Numerous strides could be made in the provision of affordable maternal healthcare to pastoralist communities in Darussalam areas of the Mudug region when organizations that support health care in Somalia and the Ministry of Health include pastoralists’ healthcare in their priorities.

## Background

Poor maternal, new born and child healthcare remains a significant problem in low- and middle-income countries (LMICs) (1). While approximately 99% of maternal deaths have occurred in developing countries, Sub-Saharan Africa (SSA) accounts for 62% of global maternal deaths (2), with 546 deaths per 100,000 live births (3). However, the maternal mortality rate (MMR) varies in SSA, with countries like Somalia having the highest MMR in the region, and with no foreseeable improvement towards achieving the Sustainable Development Goal (SDG), Article 3- to reduce the MMR to less than 70 deaths per 100,000 live births by 2030. The MMR in Somalia is 692 per 100,000, which is one of the highest in the world (4). This is because the risk factors for MMR are abundant in Somalia. The contributing factors for such a high MMR include socio-political instability and ongoing wars, as well as other sociocultural challenges, poverty, long distances to the health facility, and health system limitations (5).

The direct obstetric complications that account for the majority of maternal mortality in Africa, such as bleeding (hemorrhage), infection, hypertension, improper abortion, and obstructed labor, are all preventable (5, 6). A recent review shows that health facility-level factors have been the most frequently reported contributing factors to maternal deaths in Africa (5). The review found that contributing factors for maternal deaths by level were: (1) individual level: delay in deciding to seek help and in recognition of danger; (2) health facility level: suboptimal service delivery relating to triage, monitoring, and referral, and (3) wider health system level: transport to and between health facilities (5).

Somalia, a Sub-Saharan African country racked by civil conflict for more than three decades, has the world’s poorest access to maternal health services. While many Sub-Saharan African countries have made progress towards universal health coverage, these trends have frequently failed to reach nomadic pastoralists in particular, with little improvement since the first report on this subject was published in 2006 (7). The Somali Demographic Health Survey shows that only 24% of women in Somalia had at least four antenatal care (ANC) visits; 21% of births were delivered at a health facility; 32% of births were delivered with the assistance of a skilled health care provider; and only 11% of mothers and 10% of births had a postnatal check within the first 2 days after delivery. For the hindrances in accessing health care during pregnancy, 65% of mothers lack money to attend the health facility, more than 62% of mothers are lived far from a health facility (4).

Pastoralists are migratory people whose livelihood largely depends on livestock, with which they migrate seasonally, or episodically, in search of pastor and water. Two types of pastoralists exist in Somalia. The major group are nomadic pastoralists, who rear livestock and move with animals in search of water sources and grazing; they do not have other substances, nor do they have any permanent places of abode, but instead migrate in a seasonal manner. The number of nomadic pastoralists in Somalia is estimated at 2.3 million (8). The second group are Agro-pastoralists who engage in unspecialized herding and farming, which is primarily a mixed form of subsistence. They constitute 2.2 million, with 88% of this group concentrated in the south of the country. Nomadic pastoralists in Somalia often dwell in border areas, a highly volatile environment that is often beyond the reach of formal health services. However, the way of life of pastoral communities in Somalia has dramatically changed for the last two decades, as the current climate change has affected the amount of rain, grazing, and access to water (9), which has rendered many nomadic pastoralist families to transhumant herders who partially settle in rural villages. Although pastoralists’ way of life largely shifted from a pure nomadic life to transhumant, the government did not use this opportunity to adjust the provision of services, such as healthcare, to the pastoralist’s new way of life. Thus, on the one hand, the use of healthcare services is very limited among pastoralists (10), given several constraints stemming from their unforgiving environment, and on the other, from persistent negligence among these populations and a prevalent inequity in healthcare in the country (10). The current Somali public health services are discriminately designed for more stable and sedentary populations in urban and surrounding rural areas, ignoring remote villages and pastoralists. Therefore, while a slight progress has been observed in the access of urban populations to healthcare, particularly in major towns, pastoralist communities and transhumant have been disproportionately marginalized, making health service inequity one of the main challenges in Somalia. The aim of this study is to explore the maternal health services available for settled pastoralists (transhumant) and their families who still practice nomadic pastoralism in the Mudug region of Somalia.

### Study area

**Figure 1:**
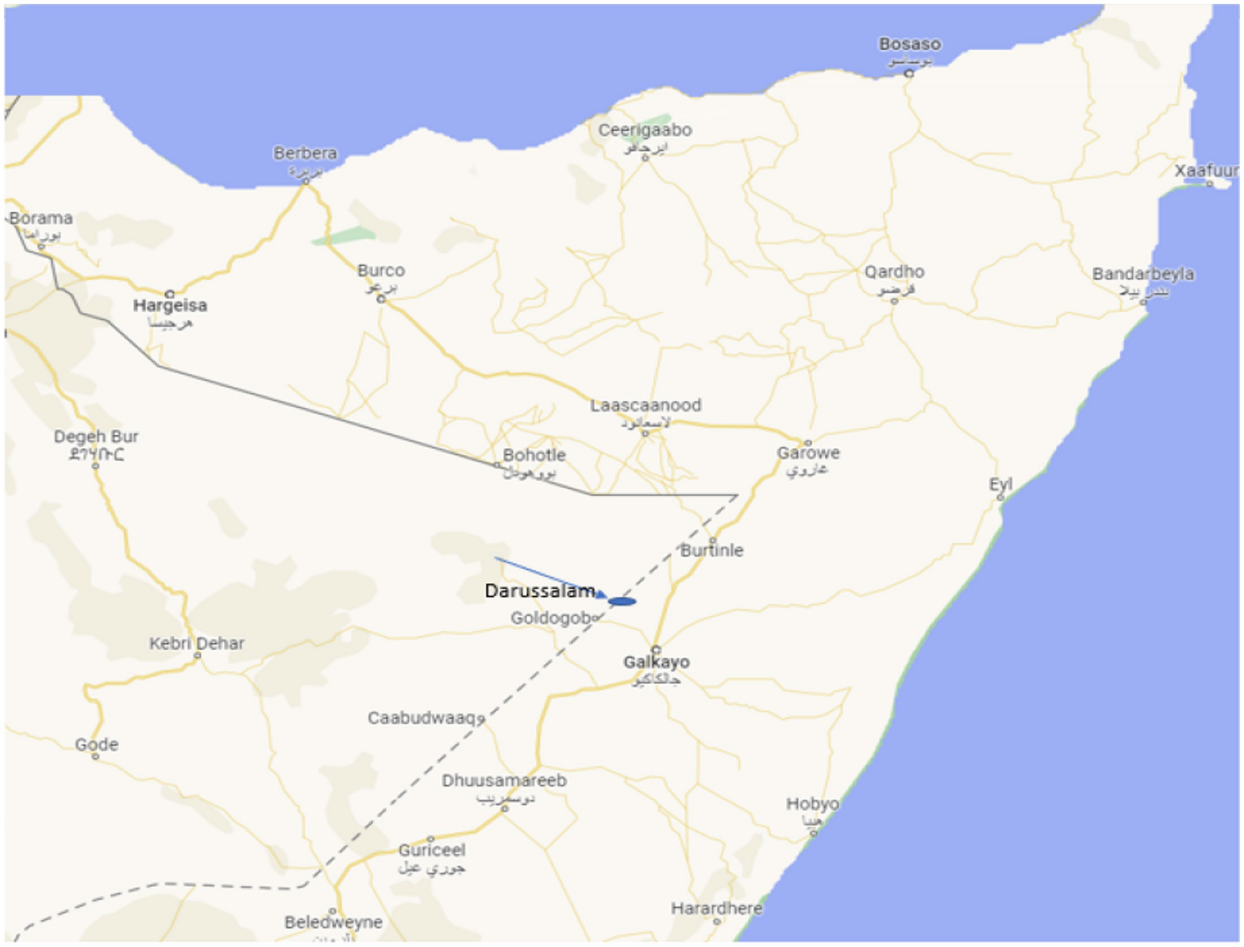
Study area.

This study was conducted in the Darussalam village of the Mudug region of Somalia’s Puntland State. It is located approximately 60 km west of the regional capital of Galka’ayo. We have chosen Darussalam due to the fact that it is a transhumant village and is located at the epicenter of pastoralist communities along the border between Somalia and Ethiopia, thereby providing health services to pastoralists on both sides of the border. The village was initiated in 2010 by predominantly transhumant, who partially gave up their nomadic pastoralist lifestyle. It is located about 40 km northeast of Goldogob town, and less than 1 km off the border of Somalia and Ethiopia (Figure 1). Darussalam and its surrounding villages accommodate about 32,000 inhabitants and provide services such as water, healthcare, and shopping to an estimated 40,000 pastoralists who inhabit an area approximately 50 km radius around the village on both sides of the border. Pastoralists in the area live in a clan system that does not recognize the national borders that separate clan members; therefore, they enjoy freedom of movement across the countries. The Darussalam area is characterized by low and erratic rainfall, high temperatures, recurrent drought, and socio-economic problems. The village has a mother-and-child health centre (MCH) as well as a private clinic that provides healthcare to both people in the transhumant villages and mobile pastoralists. The private clinic provides both inpatient and outpatient healthcare, and they charge for their services, while the MCH was built through community engagement, thus it is a public institution that provides free of charge services. The financial contribution of communities in the villages is not sufficient to cover the necessary healthcare for mothers and children in this large geographical catchment. Hence, organizations such as World Vision used to provide sporadic medical supplies to the MCH, and an occasional salary to the staff. At the time of the study, the MCH was not receiving medical supplies neither salary for its staff (Figure 1).

### Study design

We used a qualitative design, which is particularly appropriate for describing, explaining, and analyzing complex phenomena that have not been explored well. Because we needed a detailed understanding of participants experiences, both at an individual level and as a group, in-depth interviews (IDIs) and focus group discussions (FGDs) were used in collecting the data. We have chosen unstructured in-depth interviews because it is not only a flexible tool for exploring people’s experiences and their attitudes toward reality, but it is also a tool that does not impose *a priori* categorization of the questions, which may limit the field of inquiry (11). On the other hand, Focus Group Discussion is generally a tool used in health research for collecting data to inform needs assessment, evaluate services, and conduct research in group norms (11).

### Recruitment

Interviews were carried out with a total of 14 persons, including five health providers, two persons in the management of the village, and Seven community members who support the centre. In preparation for recruitment, we first contacted the chief (suldan) of the clan in the area and the commissioner of the village, to explain the objectives of the study and ask for permission. We used purposive sampling for the recruitment. Participants were recruited from different social groups, and they were selected based on their knowledge of the research topic. After we secured the admission of the chief and the commissioner, we contacted the study participants and explained the study objectives, the question guide, and their rights to participate or refuse the confidentiality of their information. The interviews and FGDs have occurred at the MCH, as per the agreement of the staff, the administration, and the chief. All participants provided their verbal consent after understanding the objectives of the study. The study was ethically cleared by the ethical committees of the Somali National University and the Somali Institute for Health Research.

### Data collection

*Semi-structured interviews* and a FGD were conducted by an experienced researcher, and administered in a quiet room at the MCH, in order to allow research participants to speak freely, and thus prevent possible response biases. After obtaining each participant’s informed consent, we started the interviews, which were tape-recorded, with each lasting between 30 and 50 min, while the FGD involved five participants and lasted for 90 min. An internal validation process was undertaken after each interview by compiling information that was not clear, which was pursued more extensively in the next interviews. This technique was suggested as a means of improving the quality and rigor of the interview guide (12). The guide consisted of open questions (Appendix 1), designed to identify the services available for pastoralists in Somalia. The first part of the interview guide focused on the socio-demographic characteristics of the participants. The subsequent sections discussed the maternal health services available, and the bottlenecks for delivering basic services to pastoralist women, including antenatal care (ANC), delivery and postnatal care (PNT).

### Analysis

The data were analyzed using thematic content analysis (13). This analytical method describes the material collected and explores its meaning to reflect what the participants said in the most objective and reliable way possible (14). Interviews were carried out in Somali and later transcribed in English. The analysis started during data transcription. To ensure that we accurately reported our participants’ own words and meanings, two authors listened to the interviews separately, and cross-checked the transcriptions to ensure they were transcribed verbatim. We initiated telephone calls to check the accuracy of the transcripts with two of the participants. The verbatim transcripts were then re-read using the audio recordings in order to validate them, and thus ensure the credibility of the responses (14). Transcripts were examined line by line to generate codes. Subsequently, we began coding and categorizing the data based on inter-coder agreements reached by the two authors. Once the coding was completed, the related codes were clustered into themes and categories. We used NVivo software (version 12) for the analysis of the data.

During the analysis, several steps have been taken to ensure the credibility and transferability of the findings. Our interview guide was piloted, and then adjusted before it was validated by the authors to ensure that it met the aims of the study. The diversification of information sources (health providers, doctors in the district hospital, village leaders and community members), and the triangulation of FGDs and in-depth interviews, also ensured the credibility of our results. Our good knowledge of the area, the culture and the language allowed us to minimize biases. Although we are aware that neutrality is impossible in qualitative research, we have undertaken an honest and reflexive approach by recognizing our biases and preventing them.

## Results

**Figure 2:**
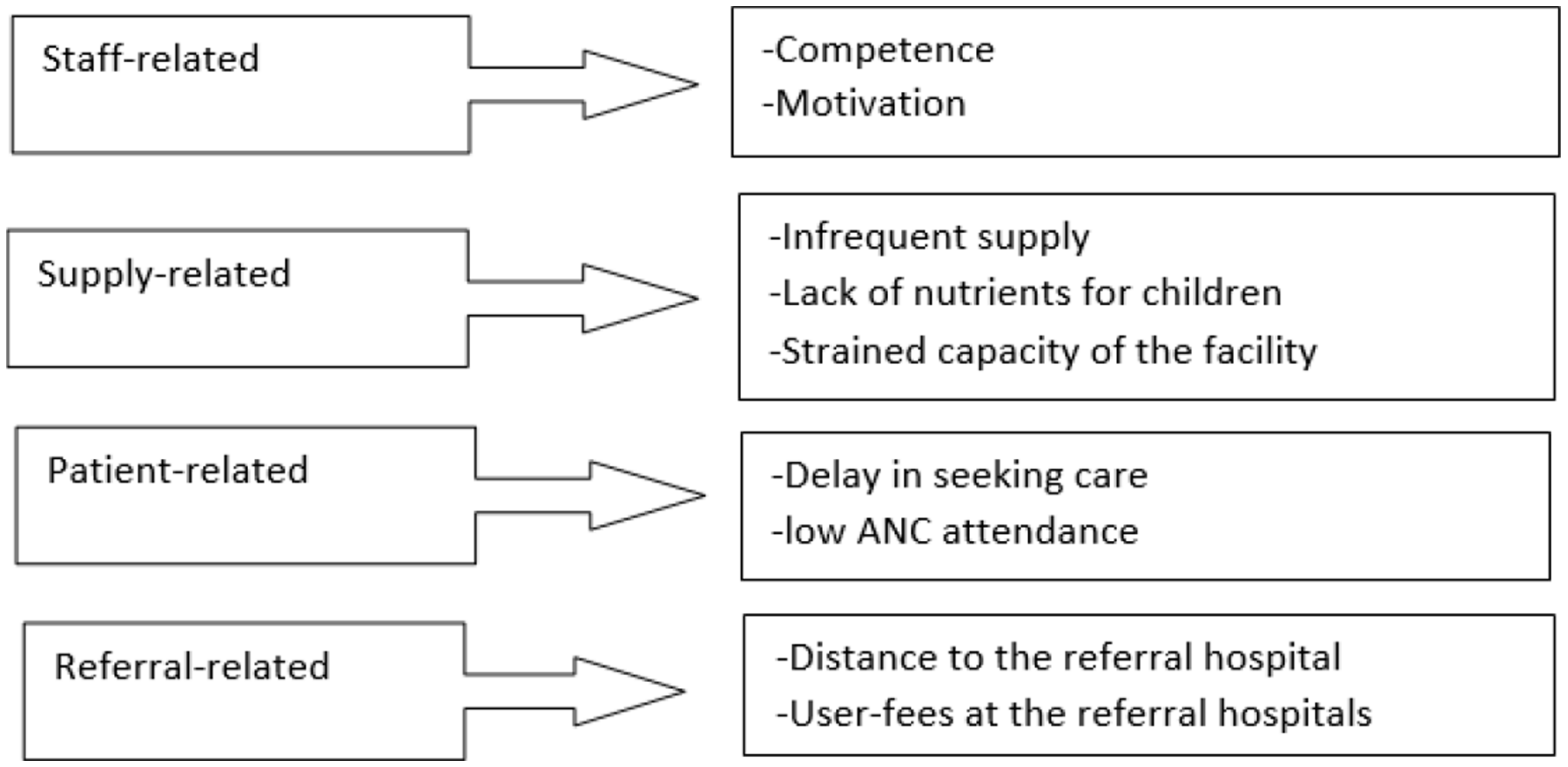
Themes and categories of challenges faced by the maternal health facilities in pastoralist areas.

The study participants reported that the facility provides maternal healthcare to pastoralist women and children living along the border between Somalia and Ethiopia for free (no user fees) for the last 12 years. These include antenatal care, delivery, postnatal care, immunization, outpatient diagnosis, and nutrition programs. Correspondingly, the facility also provides outpatient diagnostics for patients who seek other types of healthcare at the centre. Because pastoralists do not recognize national borders, the facility provides services to pastoralists and villages on both sides of the border, and that cross-border services stretched the facility beyond capacity. Study participants reported significant challenges faced by the facility. The challenges were divided into four major themes and nine categories (Figure 2).

### Staff-related constraints

#### Capacity of the staff

Participants repeatedly mentioned that the staff was a multidisciplinary team, including a health officer, a nutritionist, a nurse, a midwife, and three auxiliary ones. They also reported that they multi-tasked to minimize staff inadequacy, which might imply that the seven staff members performed more than one task during service delivery. Nonetheless, the barriers to maternal healthcare delivery include a lack of necessary training for several crucial areas, such as caesarean procedures; they have a qualified team but they lack the competence to solve some complications, such as performing a C-section and other techniques that can save mothers’ lives. According to the participants, the sustainable way to remedy these shortcomings is to strengthen the competence of the current members of the staff by offering the necessary training:

*“Our staff are clinical officers, nutritionists, midwives, and nurses. We do not have a medical doctor, let alone a specialist. We cannot manage convulsions or caesareans. We need training so that we can manage simple problems, such as performing caesareans, managing placental retention, vacuuming, and repairing tears. We also need the necessary equipment and supplies. (Health provider)”*

#### Motivation of the staff

Another barrier mentioned by participants is the remuneration of the workforce. The health staff did not receive a salary for the 6 months preceding the study period. Prior to that, they used to receive an infrequent salary for seven out of nine of the staff from the *World Vision*. According to one respondent, retaining workers is particularly challenging in rural areas of Somalia, and the lack of salary may demotivate the health staff, eventually pushing them to seek jobs in urban areas:

*“The staff of this facility is seven plus two cleaning staff. We receive a salary sometimes, and when there is no salary, we work voluntarily. In 2021, we received salary for five persons, and we shared that amount for the nine members. The salary has been paid by the World Vision. As for the year 2022, we did not get any salary at all. This brought about a lack of motivation among the staff. (Health provider)”*

### Supply related

#### Infrequent supply

Participants agreed unanimously that the supply that MCH receives from the community is generally infrequent, scarce, and not enough. Participants told us that at the moment (during the data collection) the MCH does not receive anything from either the Ministry of Health or International NGOs. The community in the village is predominantly transhumant with a low socio-economic status and according to participants, their support is neither enough nor sustainable in any shape or form:

*“The centre is supported exclusively by the community now, but their support cannot cover the needs. Everything is uncertain. Imagine that you do not know if you will receive the supplies required to provide care for an estimated 60 patients coming every day. It is a very bad situation; everything we have today is from the pocket of a view individuals here and there. It’s not sustainable. (Village management member)”*

Another participant mentioned that the MCH is lacking the vital equipment to meet the needs of women and children. These needs include an ambulance that can bring women from far away in the pastoralists’ hinterland to the MCH, and also transport women who need emergency obstetric care to the nearest referral hospitals:

*“If there is distress in the child, there is no incubator, no oxygen; if there is a need for a blood transfusion, we do not have blood bags, we do not even have the basics; sometimes we do not have essential drugs…, like, we do not have an ambulance that can bring severely ill mothers to the MCH, or when necessary, collect a competent doctor from district hospitals to solve the obstetric complications when a referral is not possible. (Health provider)”*

#### Lack of nutrients for the malnourished

A participant also narrated the prevalent malnutrition among pastoralist women, and the absence of nutritional supplements in their centre. Respondents widely agreed that, due to frequent droughts, and the low socioeconomics of the pastoral communities, pregnant women and children who seek care in this MCH are often malnourished. The demand for nutritional supplements is high, but the MCH has run out of supplies:

*“Poor nutrition is very common among pastoralist mothers, and we do not have nutritious food, supplements, or anything to help them; we can just give advice. Children come into paediatric care for malnutrition, but we have a dearth of nutrients even during droughts, during which we receive a large number of severely malnourished children. Drought cycles are frequent in this region. Now look at this child; he is from the Afar-irdood (four-gated) village, which is about 40 km away from here. If we cannot give him the care he needs, he is in great danger. He cannot even travel to the nearest district hospital. Delayed care is often why children and mothers die in here. The malnourished children used to get some nutrients, but we do not have supply at the moment. We provide other management, such as antibiotics in case of infection. (Health provider)”*

#### Strained capacity

All of the participants spoke of the mismatch between the capacity of the health centre in terms of the number of patients coming for healthcare, and the equipment and supplies they receive. A participant reported that the MCH provides healthcare to people who live in large geographical areas that are predominantly pastoralists. They pointed out that the facility is presently funded by the community with no support from the regional Ministry of Health or an international organization that supports the health system in Somalia. For that matter, the current human resources who work in the MCH lack the necessary competence, the supply is scarce, and the people who are coming for healthcare are far beyond the capacity of the facility:

*“Our health centre provides care to people living within a 50 kilometres radius, including villages such as Darusalam, Qudus, Dhamuke, Dudun,* etc.*, all of which are hamlets for settled pastoralists. The centre is stretched to give services to nearly 40 to 60 patients daily. When there is an outbreak, like cholera or measles, the number of patients increases dramatically*. *According to MOH estimates, this village and its immediate villages have a population of 32,000. Nevertheless, we provide services to people coming from over 25 pastoralist villages and nomadic areas, so you can understand the extent of the need.* (*Village management member)”*

*“I have been working in this MCH since it was established. Although it was initially a mother and-child-centre, we provide healthcare to every patient who walks in here, regardless of his or her condition. We make an estimated 40 to 41 deliveries per month*. *People in the nomadic areas on both sides of the border do not have other health facilities. The number of patients arriving poses a formidable burden, due to a lack of necessary supplies and appropriate equipment. (Health provider)”*

### Patient related

#### Poor ANC attendance for pastoralist women

Healthcare workers reported that they provide antenatal services and awareness to women in the village, but pastoralist women, who move with their herds, do not seek ANC for a reason that was not mentioned, but is obviously related to a lack of knowledge on its importance:

*“The village women often come for ANC, and we encourage them to come back for the next ANC appointment. The pastoralist women, though, come here just to give birth when they could not deliver at home, and are in a severe condition; otherwise, they do not come to the facility for either ANC or PNC. (Health provider)”*

#### Seeking care late

The participants agreed that pastoralist women often seek institutional delivery after all other traditional delivery attempts have failed. They are coming from far away; they sometimes travel hours to reach here. By the time they arrive, the situation of the mother may be severe or, at times, too complicated to be solved in the MCH:

*“Women often come to the MCH when they feel it is necessary; that is, when their situation deteriorates, and traditional midwives cannot solve the problem. The women travel a long distance to give birth here. They either come on foot or are transported by camelback if the woman is not able to walk. They arrive at the centre in a very serious condition. (Community member)”*

### Referral related

#### Long distance to the referral hospital

Participants recounted the difficulties they encountered in referring mothers to the nearest hospital in case obstetric complications arise. The referral may take a long time due to the lack of an ambulance at the MCH. This may, in the worst-case scenario, result in the death of the mother and sometimes the child. They stated that this situation can be avoided with increased staff training to be able to deal with basinc obstetric complications and the provision of the necessary equipment to the MCH, such as an ambulance:

*“When they arrive here and we understand that their situation is critical, the midwife calls the two health officers in the village: one at the MCH and the other at a private clinic in the village. We manage the minor problems at the MCH. If there is transverse or obstetric hemorrhage, we call the district hospital for help; it may take 5 h for the ambulance to arrive and take the mother to Galdogob, where she can receive emergency obstetric care. Galdogob is nearly 40 km away from here with rough roads, and the next district is Galkacyo, which is 60 km away. The mother may wait for the ambulance for a long time; sometimes the driver may refuse to travel or he is busy. The risk for the mother to die while in waiting or experiencing a life-threatening situation is high. (Health provider)”*

A health worker narrated the story of a mother and child who recently died in the MCH from a preventable cause. This tragic incident happened due to the lack of blood bags in the MCH, combined with the slowed response of the district hospital, the rough road and the distance of the district hospital. According to the participants, the availability of a blood bag alone could have saved the mother and the child:

*“We receive about five to six cases every month that require immediate referral. We cannot help even those who need a caesarean or blood transfusion because we do not have the necessary facilities. Very recently, a woman from Maaned village with obstetric hemorrhage, requiring an urgent blood transfusion, came here. We did not have blood bags for blood transfusions, nor did we have an ambulance to transfer her to the nearest district hospital. We called the Galdogob Hospital to ask them to send us a blood bag. It took time for them to respond to our request. Tragically, as the blood bag was on the way, both the mother and the child died. If we could have had an ambulance or blood transfusion facilities, we could have saved both the mother and the child. (Health provider)”*

#### User fees at referral hospitals

Some participants mentioned that the user fees constitute a formidable barrier for women to seek emergency obstetric care at district hospitals. Pastoralists are predominantly poor people who are unable to pay an expensive user fee for the C-section, blood transfusions, and similar health services:

*“We provide free healthcare services to pastoralist populations. The hospitals in the districts where we refer critically ill women, provide services that apply user fees and women have to pay for the healthcare services. The pastoralists are mostly poor; therefore, they may not be able to pay the incurred fees. In that case, when we tell them that they are being referred to the district hospital, they may sometimes say no, and consequently, we do not have a choice but to help them based on our capacity. (Community member)”*

## Discussion

In recent decades, there has been a growing interest in research activities related to the access of the maternal health of pastoralist communities. The large pastoralist community in Somalia, and the extremely limited research knowledge available in this area, justify the present study, which explores, for the first time, the constraints faced by healthcare facilities that provide maternal healthcare among pastoralist communities in Somalia. In general, the findings revealed that the maternal health facilities in pastoralist areas are poorly equipped and staffed. This implies that an estimated 4.5 million pastoralists in Somalia, who predominantly live in remote areas of the country, have been neglected from health services, especially maternal and child services. This may result in mothers and children resorting to traditional midwives and traditional healers for healthcare. A prior study on the Ethiopian side of the border found a lack of access to healthcare among Somali pastoralists (15). Consequently, 87% of pastoralist TB patients have sought traditional healthcare for their illness prior to diagnosis (16). Our findings show that the MCH in Darussalam receives approximately 40 deliveries per month, with an estimated six of those requiring referral. Without strengthening the health facilities in pastoralists’ villages, the consequences will be unnecessary, along with the avoidable deaths of mothers and children. For Somalia to progress toward the Sustainable Development Goal 3, and to reduce the staggering number of maternal mortalities, is reliant on the decision-making capacity of health policymakers in the country regarding the priority-setting of scarce resources, to help ensure that 4.5 million pastoralists in the country have access to affordable maternal healthcare. A prior review stated that ‘without adaptations to account for pastoralists’ unique subsistence patterns and cultural context, formal health services leave pastoralists behind’ (17). As pastoralists in the Mudug region, and those in many other parts of the country, are gradually adapting to a more settled lifestyle (transhumant) in their fast-evolving climate change adaptation strategy, the health service planners should adjust the services for pastoralists accordingly.

Study participants reported several barriers in the access to maternal and child healthcare among pastoralist communities. The barriers include staff-related limitations, such as an inadequate staff, and the limited competencies of the current staff to perform some of the life-saving procedures, such as a C-section. According to the WHO, an insufficient number and type of qualified health workers in remote and rural areas impedes the access to healthcare services for a significant percentage of the population, slows progress toward attaining the SDGs, and challenges the aspirations of achieving health for all (18). Therefore, the MOH and NGOs that support healthcare in Somalia ought to prioritize strengthening competencies of health workers who provide maternal healthcare to pastoralist communities. The results also show that the limited staff working in pastoralist areas receive a low and arbitrary salary that is not predictable. It is well-documented that financial incentives influence health workers’ job motivation (19). Similar to period studies (20, 21), financial and non-financial incentives, such as training opportunities and career progression, are both of importance to health workers in pastoralist areas of Somalia. A prior literature review suggested that because of the salary and career development impact on the attraction and retention of health workers in rural and pastoralist areas, there should be interventions from both government and international organizations involved in Somalia’s healthcare provisions, which include attention to the financial incentives and career development opportunities of health workers in rural areas (22).

The study findings show frequent stock-outs of nutrients for malnourished children, a shortage of essential drugs, and a lack of blood bags in case the mother requires a blood transfusion, combined with a strained capacity of the facility, which hamper the provision of reliable care to pastoralist mothers and children. In response to this mismatch, the healthcare providers tried their best to ameliorate the periodic shortage of medical supplies through borrowing blood bags, an ambulance, or a medical doctor from district hospitals. However, given the long distance between the pastoralist villages and the districts, combined with the rough roads in the area, the mother or child may die while the borrowed supplies are on the way. A prior review stated that the chronic resource strain existing at rural healthcare centers is the result of a lack of any essential resource in those areas, which may compromise a health facility’s ability to provide safe care (22). A Ugandan study underscored the importance of devising interventions aimed at improving supply chain systems in the quest to reduce medicine and other vital equipment stock-outs at health facilities providing healthcare to marginalized populations (23). Strengthening the capacity of pastoralists’ health facilities, such as the Darussalam MCH, to provide quality maternal healthcare to already marginalized communities, is the sole option to save the lives of pastoralist mothers delivering at remote health facilities.

The patient-related constraints included a low attendance by the ANC, and a delay in seeking care for pregnant women from pastoralist areas. According to the participants, pregnant women in the study areas only seek delivery care when their situation is in critical condition. In Somalia, the reasons for home delivery included financial reasons, must-use transport to reach the nearest health facility, and the fact that women are more comfortable in giving birth at home (24), which is more prevalent in pastoralist than urban areas. In line with this, a literature review stated geographic access, service quality and knowledge, and an awareness of health services as factors that influence healthcare uptake (25). The lack of necessary supplies and equipment in the study setting might also deter pregnant women from seeking care early. In line with our findings, a prior study found a number of perceived barriers to maternal healthcare utilization, including a perceived poor quality of care at facilities, such as drug and consumable stockouts, geographical barriers, and an inadequate health workforce (26). Our study found a low attendance of ANC among pregnant women. The unavailability of transportation was reported to be one of the most important barriers preventing women from accessing ANC (27), with pregnant women having to walk to the ANC facility, and having inadequate modes of transportation as key barriers to accessing care (27). In rural and pastoralist areas in Somalia, where the healthcare infrastructure is sparse, geographic access to care is a key determinant of healthcare use. Generally speaking, ANC uptake and institutional delivery are both low in Somalia (24), with pastoralist and rural women having the lowest utilization (4). It is likely that the factors that prevent pastoralist women from seeking ANC also prevent them from seeking institutional delivery until their situation reaches a crisis point (24).

Our study found referral-related constraints, which include a user fee in referral hospitals, a long distance to the referral hospital, and the lack of ambulances in pastoralists’ healthcare facilities. There is general agreement that the primary method of reducing maternal and new-born mortality rates is to increase the number of births that take place with a skilled birth attendant (SBA) present. In general, this requires the availability of basic EMOC (Basic Emergency Obstetric Care) in the facilities and a functional referral system. The pastoralist health services that we studied had no EMOC, or an ambulance to use for the emergency transfer of women who needed basic and comprehensive EMOC to the district hospital. This is the reality, despite the facility receives six cases that require an emergency referral each month. In this context, the availability of appropriate and affordable transport is therefore an essential component in efforts to reduce maternal and new-born mortality, not only in improving access to EMOC, but also in encouraging pastoralist mothers to have an institutional delivery. Conventional ambulances may be costly to purchase, but given the substantial need for emergency transport services, more affordable transport such as a Tuktuk would have improved both maternal and child health in pastoralist areas of Somalia.

In regard to user fees, in response to extremely limited public budgets for public health services, Somalia, like many other developing countries, has adopted both formal and informal systems of user fees for healthcare. In most countries, user fees seldom represent more than 15% of the total cost of hospitals and health centers, but in Somalia, where the health system is virtually privatized (28), the percentage often exceeds 60%. While hospitals rely on user fees to function, the problem with user fees in Somalia is the lack of professions to confer partial or total waivers to the poor, especially pregnant mothers and <5 children. A waiver system for the poor, particularly pastoralist mothers and children, is of critical importance in reducing maternal and child mortality in Somalia.

The study had significant limitations. The failure to generalize the findings of this study to pastoralist populations is a recognized limitation of the qualitative methods (14). We interviewed health providers, village management, and community members, but not health planners. Thus, we may have missed important information regarding the challenges faced by the health facilities. The strength of our study is that discussions and interviews took place in a very open atmosphere, and answers were not imposed through predetermined questions. Most of the views and opinions were repeatedly expressed among different individuals.

## Conclusion

Somalia’s progress toward the Sustainable Development Goals (article 3) has been stalled by the prevalent inequities in access to maternal and childcare, with this inequity being most palpable in pastoralist communities. Given that the majority of maternal and child deaths occur in rural and pastoralist areas in Somalia, the MOH and NGOs that support healthcare in Somalia ought to prioritize strengthening competencies of health workers who provide maternal healthcare to pastoralist communities. Further, the availability of appropriate and affordable transport is an essential component in efforts to reduce maternal and new-born mortality, not only in improving access to EMOC, but also in encouraging pastoralist mothers to seek an institutional delivery. Conventional ambulances may be costly to purchase, but given the substantial need for emergency transport services, more affordable transport such as a Tuktuk would have improved both maternal and child health in pastoralist areas of Somalia. Finally, a waiver system for the poor, particularly pastoralist mothers and children, is of critical importance in reducing maternal and child mortality in Somalia.

## Data availability statement

The original contributions presented in the study are included in the article/supplementary material, further inquiries can be directed to the corresponding author.

## Ethics statement

The study was ethically cleared by the ethical committee of the Somali National University and the Somali Institute for Health Research.

## Author contributions

HD and AG has initiated the research objectives, collected the data, analyzed it, and drafted the manuscript. AF, AS, and SG has participated the data collection, and took part the drafting the manuscript and reviewing the final draft. All authors contributed to the article and approved the submitted version.

## Conflict of interest

The authors declare that the research was conducted in the absence of any commercial or financial relationships that could be construed as a potential conflict of interest.

## Publisher’s note

All claims expressed in this article are solely those of the authors and do not necessarily represent those of their affiliated organizations, or those of the publisher, the editors and the reviewers. Any product that may be evaluated in this article, or claim that may be made by its manufacturer, is not guaranteed or endorsed by the publisher.

## Appendix 1

Question guide

Demographics

Gender

Occupation

Education

Main questions and overview of follow-up questions (FGDs and interviews)

1. Can you explain about the population in this area?
2. Can you tell me about the health care facilities available in this area? Type\number\services provided\private\ public.
3. Can you explain the beneficiaries of the health care facilities you mentioned?
4. How does the maternal and child health centre (MCH) work? Services\the staff qualifications, competencies, motivations\ financing\the supply?
5. What are the constraints and limitations that the MCH faces as regards to provision of acceptable health care to the women and children in the area? And how do you think that these constraints can be solved\overcome?
6. What else do you want to add?
